# Top-income adjustments and official statistics on income distribution: the case of the UK

**DOI:** 10.1007/s10888-022-09532-y

**Published:** 2022-04-01

**Authors:** Stephen P. Jenkins

**Affiliations:** 1 Department of Social Policy, London School of Economics and Political Science, London, WC2A 2AE UK; 2 IZA, Institute of Labor Economics, Schaumburg-Lippe-Strasse 5–9, 53113 Bonn, Germany

**Keywords:** D31, C81, Inequality, Income inequality, Top incomes, Tax return data, Survey data, Data combination, SPI adjustment

## Abstract

UK official statistics on income distribution have incorporated top-income adjustments to household survey data since 1992. This article reviews the work undertaken by the Department for Work and Pensions and the Office for National Statistics, and the academic research that influenced them, and reflects on the lessons to learn from the UK experience.

**JEL Classification Codes** D31 · C81

## 1 Introduction

Over the last two decades, researchers have made substantial advances in our knowledge of top incomes, and of the nature and quality of information about them provided by household survey and personal income tax administrative data, as this special issue testifies. Researchers now commonly acknowledge the problem of survey under-coverage of top incomes and have proposed a range of methods to address them. However, national statistical agencies do not typically incorporate top-income adjustments when they produce survey-based income distribution data and the reports from which most research users learn what has been happening to inequality. Thus, there is a data quality disconnect between academia and most official statistical monitoring of income inequality. One notable exception concerns the United Kingdom (UK) whose official income distribution statistics have incorporated top-income adjustments for almost thirty years. The aim of this article is to summarize the work

✉ Stephen P. Jenkins
s.jenkins@lse.ac.uk

1 Department of Social Policy, London School of Economics and Political Science, London WC2A 2AE, UK

2 IZA, Institute of Labor Economics, Schaumburg-Lippe-Strasse 5–9, 53113 Bonn, Germany undertaken by the Department for Work and Pensions (DWP) and the Office for National Statistics (ONS), and the academic research that influenced them, and to reflect on the lessons to learn from the UK experience.

In the next three sections, I describe three streams of work, starting with DWP's pioneering top-income adjustment approach that produces the data underpinning their annual report on income distribution, *Households Below Average Income* (*HBAI*).[1] DWP (2021*a*) is the latest edition. The same top-income-adjusted data are also the basis of the Institute for Fiscal Studies' much-cited annual commentaries on inequality and poverty (see, e.g., Cribb et al. 2021), and many other researchers use the data as well because they are made available in unit record form through the UK Data Service. Second, I summarize the critique by Burkhauser, Hérault, Jenkins, and Wilkins (Burkhauser et al. 2018a; Burkhauser et al. 2018b), hereafter BHJW, of the DWP's top-income adjustment. I proceed to discuss BHJW's refinements of it and the estimates of inequality levels and trends that result from their approach. In both streams of work, the household survey is the Family Resources Survey (FRS) and the top-income adjustments draw on information from income tax administrative records contained in the Survey of Personal Incomes (SPI).[2] Third, I discuss the ONS's recent work (2019; 2020a) which combines information from the Living Costs and Food Survey (LCFS) with SPI data. This work drew on BHJW's research explicitly and is the foundation of the new ONS income inequality series (ONS 2021a). In Section 5, I consider the combination of circumstances that led to the introduction of the UK's top-income adjustments by the DWP and the ONS and discuss prospects for introductions by other countries.

## 2 The DWP's SPI-Adjustment

The DWP introduced its top-income adjustment to survey incomes, the 'SPI-adjustment', in 1992. In an early methodological review, the DWP stated that the goals of its approach were '[t]o improve the quality of data on very high incomes and combat spurious volatility' (Department of Social Security 1996: 23). The latest edition of the *HBAI* Methodology report reiterates the dual role. It tells data users that 'High incomes' are one of the 'Issues to Consider':

> Comparisons with Her Majesty's Revenue and Customs' Survey of Personal Incomes (SPI), which is drawn from tax records, suggest that the FRS under-reports the number of individuals with very high incomes and also understates the level of their incomes. There is also some volatility in the number of high income households surveyed. Since any estimate of mean income is very sensitive to fluctuations in incomes at the top of the distribution, an adjustment to correct for this is made to 'very rich' households in FRS-based results using SPI data. (DWP 2021b: 12).

Beyond general statements like these, the SPI-adjustment is poorly documented, motivating BHJW (2018*a*) to compile as comprehensive an understanding of it as possible. BHJW confirm that that the SPI-adjustment reduces the volatility of top incomes but also argue that

[1] The title reflects the DWP's principal responsibility in official statistics, for poverty estimates. Despite the title, the reports and associated datasets cover the whole of the income distribution, not simply the lower ranges.
[2] See BHJW (2018*a*, *b*) for discussion of the FRS and SPI datasets.

this could be achieved using a more parsimonious method. In this article, I focus on the data quality aspect, top-income under-coverage.

There are five key steps in each year's SPI-adjustment, as follows (see BHJW 2018*a* for further details):

1) *Align the household survey data with the SPI data* This has two elements: (i) restrict the survey and SPI samples to individuals aged 15+ ('adults'), and (ii) for each adult, use the detailed survey unit record data on income components to construct a measure of individual gross income. Gross income is total taxable income from the market plus taxable government transfers (i.e., prior to the deduction of income tax).
2) *Identify 'very rich' individuals in the survey* A respondent is 'very rich' if his or her income is above a threshold. There are four thresholds, depending on whether the respondent is a pensioner or non-pensioner and on whether the respondent resides in Great Britain (England, Wales, and Scotland) or Northern Ireland. (The two binary classifications characterise four top-income groups.) The thresholds are set at a level above which, for each group, the survey responses are considered 'volatile due to the small numbers of cases' (Department for Work and Pensions 2016: 20). Over time, the main change in procedure has been to adjust incomes for a fixed fraction of the population rather than for all individuals above a specified income threshold, the aim being to prevent adjustment of an increasing fraction of observations. Since 2009/10, the target has been to adjust at most around ½ per cent of (weighted) survey observations overall.
3) *Impute SPI income-group means to the survey 'very rich'* The income of each 'very rich' respondent in the FRS is replaced by the mean income of a corresponding group (as defined above) of 'very rich' individuals in the SPI data. A distribution with four mass points replaces the income distribution reported for the 'very rich'.
4) *Recalibrate the survey weights* The SPI provides estimates of the numbers of 'very rich' individuals in the population. Using a well-known calibration weighting methodology, DWP statisticians recalculate the survey weights (grossing factors) using the numbers of high-income pensioners and non-pensioners as control totals in addition to the many other control totals used to derive the survey grossing-up factors applied to non-SPI-adjusted data. The recalibrated weights are little different from the original weights.
5) *Recalculate measures of household income using the revised individual-level data*. The final stage recomputes the household income measure. UK official statistics assesses individuals' material living standards in terms of the equivalized net (disposable) household income of the household to which they belong (the same 'Canberra Group' (2011) definition as used by Eurostat and the OECD). Net income is gross income from all sources less payments of income tax, employee national insurance contributions, local taxes, and some other deductions. Net incomes are equivalised using the modified-OECD equivalence scale.

Clearly, the SPI-adjustment approach attempts to address issues of top-income-related item under-response (via imputations from the SPI data at Step 3) as well as unit non-response (via recalibration of the survey weights at Step 4).[3]

[3] Although in principle the recalibration of the survey weights may also help address top-income-related item under-response, the effect is likely to be small. See also footnote 4 on related issues.

## 3 BHJW's critique of the SPI-Adjustment, and their SPI2-adjustment

BHJW's analysis for the UK was motivated by a desire to reconcile two sets of estimates of income inequality trends. On the one hand, the DWP and ONS survey-based estimates, typically summarised by the Gini coefficient for equivalized net household income, suggested that inequality had changed hardly at all since the start of the 1990s. On the other hand, estimates of top-income shares from the World Inequality Database derived from SPI data suggested a distinct inequality increase since the 1990s (BHJW, 2018*b*, Fig. 1). BHJW noted that some of the differences in inequality trends might arise because the data series used different definitions of income and income-receiving unit, and different inequality indices, but recognised that differences reflect survey top-income under-coverage as well.

BHJW's work for the UK built directly on team members' earlier work for the USA for which top-income under-coverage was also a key issue. For example, Burkhauser et al. (2012) showed that estimates of the top 1%'s income share derived from the Current Population Survey (CPS) fell below corresponding estimates derived from Internal Revenue Service personal income tax data, even when using common income definitions and adjusting the survey estimates upwards to account for CPS top-coding. Atkinson, Piketty, and Saez (Atkinson et al. 2011) cited these results in their review of problems with surveys, arguing that income tax data provide a better view of the top income ranges.

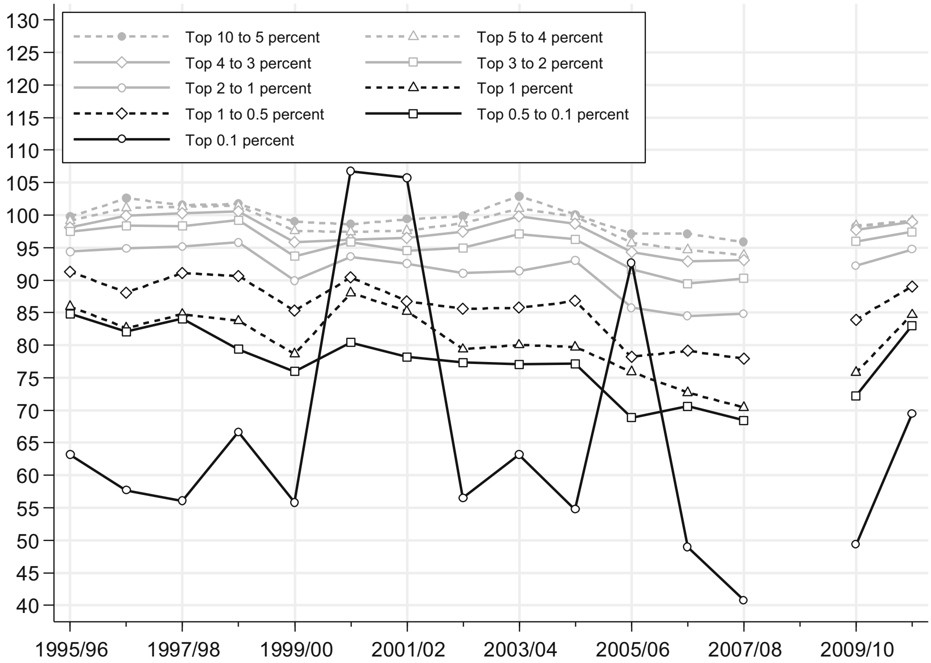

**Fig. 1** Survey under-coverage of top incomes, by income group and year. Source: Burkhauser et al. (Burkhauser et al. 2018b, Fig. 3), based on FRS and SPI data. Notes: Percentages below 100% indicate survey under-coverage of top incomes: see main text. Northern Ireland is included only from 2002/03 onwards. There are no SPI data available for 2008/09.

An important first step in BHJW's UK work was therefore to examine the nature and extent of survey under-coverage of top incomes, having first aligned the FRS and SPI data sources in the sense discussed earlier. For each year, BHJW took survey-based top-income quantile groups and their tax-based counterparts and compared group mean incomes. A ratio of survey mean to SPI mean below 100% represents survey under-coverage. Clearly, analysing under-coverage by comparing quantile group means in each source assumes that the under-coverage problem is income-related under-reporting (partial item response) rather than unit non-response. BHJW claim that this assumption approximates the situation for the FRS and provide several supporting reasons.[4]

Figure 1 shows that FRS under-coverage of top incomes is a serious problem. Under-coverage is particularly acute at the extreme top of the distribution (at the 99th percentile or above). There are ratios of 90% or smaller. This is especially so for the top 0.1%, but here the few respondents underlying the survey-based estimates lead to substantial year-on-year volatility as well. However, under-coverage also occurs further down the distribution, especially in the mid- to late-2000s. Importantly, it occurs below the DWP's SPI-adjustment range (approximately the top ½% in recent years). Under-coverage is much less of a problem for the 'top 10% to 5%' group for most of the period (ratios around 100%), though more so later.

BHJW's (2018*a*) analysis led them to four conclusions. First, to properly address top-income under-coverage, you need to adjust more than the SPI-adjustment does (at most ½%), i.e., around 3% to 5% of the top incomes. Second, you need to take better account of the inequality within the very rich group: under-coverage is greater at the very top than in top ranges below the very top. This suggests that the cell mean imputations at the top should be more granular, using a greater number of values than the SPI-adjustment does and varying more by income position. Third, and related, BHJW argued that the four stratification groups used in the SPI-adjustment were unnecessary because the defining characteristics were poor markers of top-income status. Fourth, BHJW observed that the SPI cell means used at Step 3 of the SPI-adjustment are derived using projections from SPI data for a financial year at least one year, sometimes two years, prior to the FRS year. (Current year SPI data are unavailable when the DWP prepare their income distribution series.) BHJW showed that this could introduce systematic biases and recommended further work on this topic.

In BHJW's (2018*b*) article, they practiced what they preached: building on their first three conclusions, they implemented an 'SPI2' adjustment using yearly FRS and SPI data for the period 1995/96 through 2010/11, the latest year for which data were available when the research began. Step 1 (data alignment) for the SPI2-adjustment is the same as for the SPI-adjustment.

Steps 2 and 3 are substantively different. In terms of how far down from the top to adjust (conclusion 1), BHJW (2018*b*) considered variants with adjustments made to the incomes of the top 10, 5, 4, 3, 2, 1, or ½%. They focused attention on the 5% adjustment because it

[4] For example, using the same reconciled data as BHJW (2018*b*), Jenkins (2017, Appendix I) shows that the proportion of (weighted) observations above the real income value corresponding to *p*99 in the FRS data is close to 0.01 in the SPI data for many years. There are several explanations why unit non-response plays a relatively minor role. For example, the FRS survey weights may be doing a relatively good job of adjusting for top-income-related unit non-response: they use external information not only about demographic characteristics but also characteristics related to income such as housing tenure and council tax band. (This is unlike the US Current Population Survey, for which income-related non-response is analysed by Korinek et al. 2006 and Morelli and Muñoz 2019.) Also, there may be problems with the UK tax data which mean that it is not only survey data that do not capture the very highest income earners (i.e., both data sources may identify the same top 1% once problems with both sources are acknowledged). See Burkhauser et al. (2020) for further discussion.

addressed under-coverage and minimized discontinuities in density between the imputed top income distribution and survey incomes for non-top incomes ('seam' effects), while also being a suitable compromise given the changes in under-coverage over the entire period. BHJW (2018*b*) found that SPI2-adjusted estimates were insensitive to the choice of variant as long it included at least the top 2%. Another major difference from the SPI-adjustment concerned granularity – how many cell means to use within the fraction of top incomes adjusted. BHJW divided the (weighted) adult population, ranked by individual gross income, into 1000 equal-sized groups separately for the SPI and the survey; then they calculated mean income for each of the SPI permille groups; and imputed these group means to the corresponding survey permille group. For example, in the variant adjusting the top 5% of survey incomes, BHJW replaced the individual-level survey income in each of 50 groups with the mean income of the corresponding group in the SPI.

Figure 2 illustrates the SPI2-adjustment in action and contrasts it with the SPI-adjustment. It shows kernel density estimates of the upper tail of three distributions for 2010/11 – unadjusted survey data (labelled 'HBAI'), SPI-adjusted data (labelled 'HBAI-SPI'), and SPI2-adjusted data using the variant adjusting the top 5% of survey incomes (labelled 'HBAI-SPI2). The dashed vertical lines mark the unadjusted survey distribution's $90^{\text{th}}$, $95^{\text{th}}$, $99^{\text{th}}$, and $99.5^{\text{th}}$ percentiles. The unadjusted distribution (grey density line) has a long right-hand tail with a very tiny amount of density mass. The DWP's SPI-adjustment replaces the sparse upper tail in the HBAI distribution with four clumps of density mass: look at the dashed density line. Only two clumps, for the 'very rich' non-pensioner groups, are visible; the effect of changing the incomes of individuals in the two 'very rich' pensioner groups is imperceptible. By contrast, the SPI2-adjustment places density mass over a greater income range than the SPI-adjustment and the concentration in the top income range is greater in total: look at the solid black line. The reduction in sparsity of coverage of top incomes by the SPI2-adjustment also reduces the chances of non-robustness in inequality estimates due to 'high leverage' outliers in the survey data (Cowell and Flachaire 2007) as well as decreasing the likelihood of spurious volatility in estimates of inequality trends – one of the DWP's concerns.

Steps 4 and 5 also differ for the SPI2-adjustment by comparison with SPI-adjustment. BHJW (2018*b*) were unable to recalibrate the weights using the more detailed SPI top income group numbers because they had no access to the DWP's calibration weight software and other control totals. Instead, their estimates are based on the recalibrated SPI-adjustment weights. This is likely to be a relatively minor issue because the DWP's recalibration leads to only very small changes in weight values and distribution. Another difference arises at Step 5. BHJW (2018*b*) recalculated household incomes using the SPI2-adjusted individual gross incomes, and also equivalized them using the modified-OECD scale. However, they had no access to tax-benefit microsimulation software for deriving net incomes for multiple years. Thus, BHJW focus on estimates of inequality levels and trends in gross incomes.

BHJW (2018*b*) show that UK (gross) income inequality is greater when calculated using SPI2-adjustment than when using DWP's SPI-adjustment, and that inequality increased after the mid-1990s to a greater extent.[5] The increases in inequality levels are unsurprising because

[5] Jenkins (2017) derives very similar results using the same data sets but using a semi-parametric approach: he estimated inequality among top survey incomes by fitting generalized Pareto (Type II) distributions to SPI top incomes.

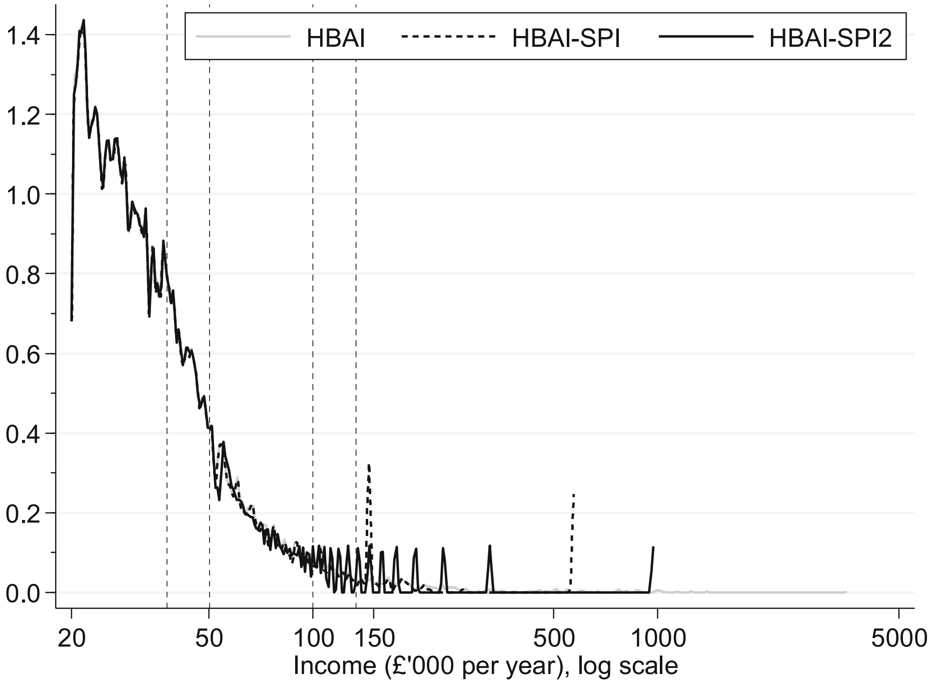

**Fig. 2** Density estimates of the upper tails of unadjusted and adjusted distributions of individual gross income, 2010/11. Source: Burkhauser et al. (2018b, Fig. 4), based on FRS and SPI data for 2010/11. Notes. Density estimated for incomes greater than £20,000 per year using an Epanechnikov kernel and bandwidth of 0.008. The dashed vertical lines show the 90th, 95th, 99th, and 99.5th percentiles in the unadjusted FRS data (labelled 'HBAI'). The SPI-adjusted data are labelled 'HBAI-SPI' and the SPI2-adjusted data are labelled 'HBAI-SPI2'.

the SPI2-adjustment attributes more income to the top end of the income distribution. For example, BHJW estimate the share of total gross income held by the top 1% in 2010/11 to be 12.9% when the SPI2-adjustment is applied but 11.6% using the SPI-adjustment (BHJW 2018*b*, Fig. 6 and Appendix Table E1). Table 1 reports, for selected years prior to Great Recession onset, estimates of the Gini coefficient and three other indices – mean logarithmic deviation (MLD), Theil, and half the squared coefficient of variation (HSCV). These are generalized entropy indices with sensitivity parameters 0, 1, and 2 respectively. The larger the parameter, the more sensitive is the index to income differences at the top of the distribution relative to those in the middle or at the bottom – the MLD (and Gini coefficient) are middle-sensitive indices.

The Gini coefficients for the SPI2-adjusted distributions are larger than the corresponding Ginis for the SPI-adjusted distributions, as is the percentage change between 1995/96 and 2007/08 (10% versus 7% respectively). For the other three indices, and each year, the level of inequality is also larger in the SPI2-adjusted data. Moreover, the more top-sensitive the index, the larger is the measured inequality increase. For example, the increase between 1995/96 and 2007/08 in the SPI2-adjusted HSCV is much larger than in its SPI-adjusted counterpart (141% versus 52%). For the less top-sensitive MLD, the increases are 24% and 16% respectively. BHJW (2018*b*) also show that, regardless of the index chosen, most of the increase in inequality over the period 1995/96 to 2007/08 occurred between 2004/05 and 2007/08.

**Table 1** Inequality index estimates, by year (SPI2 method)

| Inequality index | Adjustment | Level | | | | Percentage change |
|---|---|---|---|---|---|---|
| | | 1995/96 | 2001/02 | 2004/05 | 2007/08 | 1995/96 to 2007/08 |
| Gini coefficient | SPI2 | 0.380 | 0.400 | 0.390 | 0.418 | 9.8 |
| | SPI | 0.375 | 0.394 | 0.384 | 0.399 | 6.6 |
| Mean logarithmic deviation (MLD) | SPI2 | 0.245 | 0.275 | 0.261 | 0.304 | 23.9 |
| | SPI | 0.239 | 0.268 | 0.254 | 0.277 | 16.1 |
| Theil index | SPI2 | 0.275 | 0.333 | 0.317 | 0.396 | 44.1 |
| | SPI | 0.266 | 0.325 | 0.299 | 0.332 | 24.8 |
| Half squared coefficient of variation | SPI2 | 0.486 | 0.778 | 0.721 | 1.170 | 140.5 |
| (HSCV) | SPI | 0.470 | 0.765 | 0.619 | 0.705 | 49.8 |

Source: extract from Burkhauser et al. (2018b), Table 3. Inequality indices refer to distributions of gross household income, equivalized using the modified-OECD scale. The individual is the unit of analysis, with estimates based on the full private household population (aged less than 15 as well as aged 15+)

## 4 The ONS top-income adjustments and revised income distribution series

In 2018, the ONS published a wide-ranging plan for work under the heading of transforming household finance statistics. Referring to one of the projects, they stated that:

> [i]n line with research into using administrative data to tackle potential under-reporting of high-income earners in surveys, (for example, Burkhauser et al. (2018a)), we are planning to prioritise the development of an adjustment for the income of high earners in the next year. We will work closely with DWP on this research and learn from their experience in using an adjustment for high earners using HMRC Survey of Personal Incomes (SPI) data in the Households Below Average Income release.' (ONS 2018: 4.)

The subsequent methodological work resulted in two methodological reports (as well as revised data and statistical series, discussed shortly). The first report (ONS 2019) states that they build on the work of BHJW and they derive headline series in an almost identical manner to BHJW (2018*b*) as described in the previous section. The main difference is that the survey used is the Living Costs and Food Survey (LCFS), with fewer respondents than the FRS (around 5000 households per year rather than 20,000) but an essential input into the ONS annual income distribution report *Effects of Taxes and Benefits on Household Incomes* (see, e.g., ONS 2021b). In addition, the ONS derived equivalized household net incomes (as the DWP do) and used more up-to-date data than BHJW (through to 2017/18).

In line with the points made in BHJW's critique of the SPI-adjustment, the ONS (2019) report documents top-income under-coverage in the LCFS in the same way as BHJW, and gives specific attention to the issues of how far down from the top of income distribution to make adjustments (considering variants of 5, 4, 3, 2, and 1%) and the number of income groups within this range (the granularity issue; the ONS consider group sizes of 0.25, 0.5, and 1%). Interestingly, the ONS show that the different variants lead to almost identical estimates of the Gini coefficient (ONS 2019, Figs. 2 and 5), each of which is between one and four percentage points higher than the Gini for unadjusted data depending on the year. The adjusted series also indicate different inequality trends: the unadjusted data show a decline in the Gini coefficient between 2010/11 and 2015/16 whereas the adjusted series shows a rise.

ONS continued their methodological research, summarizing the completed work in a substantial report released in February 2020 (also written up as Webber et al. 2020). The most important innovation compared to their 2019 work was introduction of a variation on BHJW's SPI2 approach, with differences at Steps 2–4. The new approach defines top-income quantile groups in the SPI as before (with specific choices about how far down the distribution the adjustment is made and about granularity). However, they define income groups in the survey data using the income values that characterize the SPI quantile groups (i.e., not the income values that characterize the within-survey quantile groups, as before). For each SPI income group, the group mean income replaces the income of each member of the corresponding survey income group. At a further step, the alternative adjustment method reweights the survey income groups so that their weights are the same as the corresponding SPI quantile groups and also reweights the unadjusted portion of the survey data to maintain the same overall population totals for each weighting variable. ONS label this new adjustment method the 'reweighting' approach to distinguish it from the 'quantile' approach that they and BHJW had used earlier. 'Income replacement with reweighting' and 'income replacement', respectively, would be clearer and more accurate labels.

The ONS argues that '[w]here the primary challenge affecting top incomes is that of under-reporting rather than lower survey participation of the richest households, the effects of the two methods should be largely equivalent in practice. However, where lower participation also has a significant impact, the second "reweighting" method should prove more effective' (2020a: 7). I agree that in principle recalculation of the weights following top-income group income replacement is likely to be an improvement over income replacement alone. Observe too that having the two stages is analogous to what the DWP SPI-adjustment does at its Steps 3 and 4, a point returned to later.

The ONS (2020a) report proceeds to compare estimates for the two methods while, at the same time, re-examining the issues of the top-income threshold and granularity within the top-income range. They show time series of estimates of the average income of the top 10% and of the top 1%, and the Gini coefficient for each of the reweighting and quantile methods, side by side.[6] Balancing a number of considerations, the ONS plump for an adjustment based on the top 3% of individuals (a '97% threshold') and for using quantile band widths of 0.5%.

As for the 'beauty contest' between the reweighting and quantile methods, the ONS charts show a remarkable similarity in corresponding estimates. Figure 3 shows estimates for the Gini coefficient according to both adjustment methods, together with Gini estimates based on unadjusted LCFS data. The two adjusted data series are very similar in terms of levels and trends and, as expected, are greater in magnitude than the unadjusted data Gini, by up to three percentage points towards the end of the period. The quantile and reweighting estimates for a

[6] Additionally, the ONS examined BHJW's (2018*a*) recommendations to not have different thresholds for strata distinguished by region or age, and to check whether using projected SPI means (rather than actual outturns) led to systematic biases. The ONS decided to make separate adjustments for pensioners and non-pensioners on the grounds that 'an adjustment applied just to the overall distribution would be unlikely to fully adjust for under-coverage of the incomes of pensioners' (2020*a*: 8), that breakdowns between the two groups were important for their statistical analysis, and that this stratification would maintain consistency with the DWP's approach. The ONS also concluded that their analysis provided no compelling case for revising measures of Gini coefficients once final SPI data are made available (though they would continue to monitor the situation). In support of this, they show that 'the impact of moving from projected to final data leads to, on average, a 0.2 percentage points revision of the Gini coefficient' and argue that the 'the 95% confidence intervals of published Gini coefficients are usually wider than even the largest observed revision' (2020*a*: 8).

given year never differ by more than half a percentage point (except in 2007/08) and mostly by less.[7] This indicates that item non-response (top income under-reporting) contributes far more to LCFS top-income under-coverage than unit non-response does – as BHJW suggested for the FRS.

Nonetheless, the ONS noted that some systematic differences are perceptible and these, as well as some other reasons, swung them in favour of the reweighting method:

> That the Gini coefficient is marginally higher under the reweighting approach suggests that non-response at the top of the distribution does play some role. This indicates that although more complex, the reweighting approach is preferred. Another reason for adopting the reweighting approach comes from Fig. 2 [not shown here], which highlighted that, although nonresponse may be a lesser concern for the overall income distribution (mirroring the findings of Burkhauser et al.), there is evidence to suggest it may be more noticeable in the distribution of pensioners' incomes. A further important consideration is coherence. The reweighting approach is closest in methodological terms to the SPI adjustment currently used by the DWP's HBAI statistics. Adopting this approach therefore ensures coherence in terms of methods across the UK Government Statistical Service (GSS). (ONS 2020a: 14.)

ONS therefore chose to proceed with the reweighting approach in their production of subsequent official series, with statistics such as the Gini coefficient recalculated using the new approach for each year starting from 2001/02.

There has been a further innovation subsequently, also arising from the *Transformation of ONS Household Financial Statistics* project (ONS 2018). Estimates for the most recent years are from a new data source, the Household Finances Survey (HFS), which is a combination of the LCFS and the Survey on Living Conditions, producing a sample of around 17,000 private households (ONS 2020b) The principal impact of the larger sample size on ONS's income distribution statistics is greater precision. For 2018/19 the 95% confidence interval for the Gini coefficient falls from 4.5 percentage points using LCFS data to 2.5 percentage points using HFS data, and the HFS-derived Gini coefficient is slightly higher than the LCFS one reflecting a slightly higher average income in the richest fifth (ONS 2020b: 7).

Fig. 4 shows ONS's most recently published Gini estimates (ONS 2021a), based on the reweighting method, and extending to 2019/20 (series HII).[8] To provide additional context, I extend the ONS series back to the earliest year for which an estimate is available (1977), and show the DWP's Gini estimates, as reported in *HBAI*, for the same years (series HBAI). The estimates for 1977 and 1978 provide an important reference point, as they are the lowest Ginis

[7] The changes between 2007/08 and 2010/11 may reflect the Great Recession. However, behavioural response by high-income taxpayers complicates interpretations. The UK introduced a 50% top marginal rate of income tax in April 2010 (up from 40%), and the announcement and introduction of this tax rate provided incentives for high income taxpayers to bring forward income to 2009/10 that would otherwise have been reported in 2010/11 income tax returns or possibly later years. This is the process of 'forestalling'. Subsequently, a reduction in the top marginal tax rate to 45% provided incentives to defer income to later tax years ('reverse forestalling'). See Seely (2014) for further discussion.

[8] The HII Gini estimates shown in Figure 4 are larger than the weighting approach estimates shown in Figure 3, although the ONS derived both sets using the same definitions of threshold (97%) and quantile bandwidth (0.5%). The reason is that the ONS revised their derivation code between reports, fixing a few bugs along the way, and the effects of the revisions were greatest for the earlier period (ONS Household Income and Expenditure team, personal email communication, 30 July 2021).

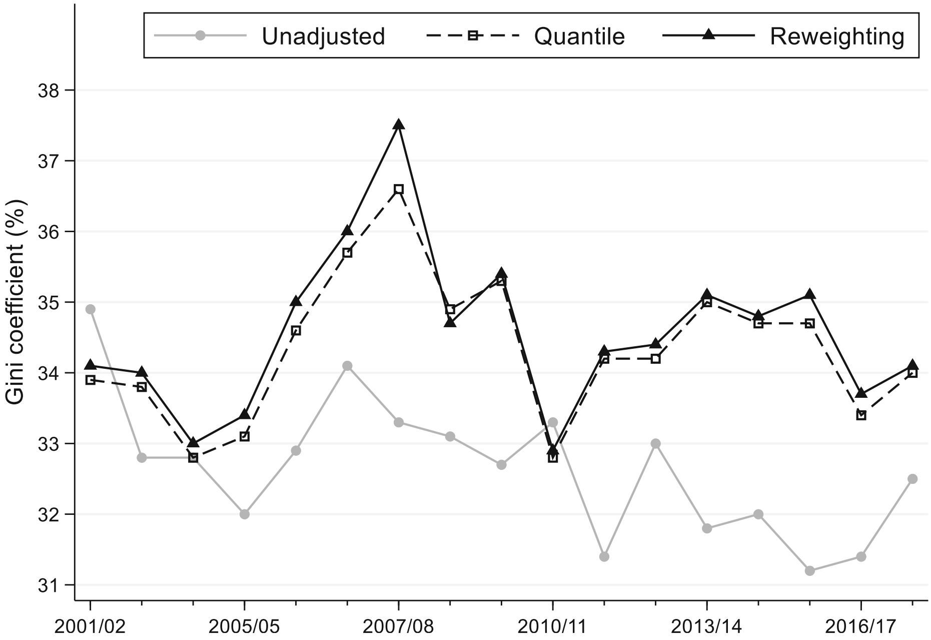

**Fig. 3** Gini coefficients, by ONS adjustment method. Source. spreadsheets accompanying ONS (2020a). Notes. All distributions refer to distributions of net household income, equivalized using the modified-OECD scale. The individual is the unit of analysis, with estimates based on the full private household population. The unadjusted estimates are based on LCFS data. The quantile and reweighting estimates are based on LCFS data with a top-income adjustment using imputations from the SPI employing a 97% threshold and 0.5% quantile band width (see main text).

observed since 1961.[9] The vertical dashed lines indicate the survey years for which the DWP and the ONS first used their top-income adjustments and the year the ONS switched to using HFS data.

The HII and HBAI series provide similar estimates of Gini levels and trends in a broad-brush sense: both show the substantial rise in inequality during the 1980s and the smaller rise combined with fluctuations since the start of the 1990s. However, there are some systematic differences between the series. First, before the early-1990s, when the Family Expenditure Survey was used by both the ONS and the DWP, the HII Ginis were almost always slightly larger than the corresponding HBAI Gini. It is unclear why. Second, the HII series exhibits greater variability than the HBAI series since the mid-1990s. This is because the sample size of the yearly LCFS (and its predecessors) is substantially smaller than the FRS's, as explained earlier. When the HFS is used to produce more yearly estimates in future, presumably the differences in variability between HII and HBAI series will decline.

Third, over the period when the HBAI series includes a top-income adjustment but the HII series does not, i.e., 1992 to 2001/02, the HBAI series lies above the HII one (in all but one year), which is what one would expect. Fourth, after 2001/02, i.e., the years for which the ONS

[9] The Institute for Fiscal Studies extend the HBAI series back to 1961: see the spreadsheet accompanying Cribb et al. (2021).

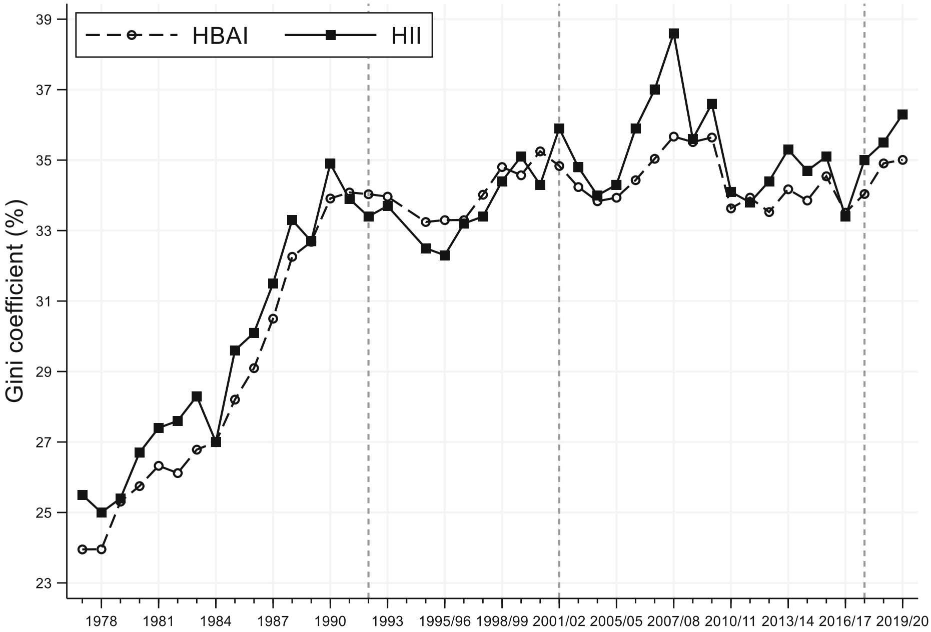

**Fig. 4** Gini coefficient series: ONS 'HII' and DWP 'HBAI' estimates. Source. spreadsheets accompanying ONS (2021a) for HII series and Cribb et al. (2021) for the HBAI series. Notes. All distributions refer to distributions of net household income, equivalized using the modified-OECD scale. The individual is the unit of analysis, with estimates based on the full private household population. The HBAI estimates are the same as published by the DWP and are based on the FRS (and Family Expenditure Survey before 1994/95). Northern Ireland data included from 2002/03 onwards. The HBAI series uses the SPI-adjustment method from 1992 onwards. The HII series is based on data from the LCFS (and its predecessors) up to 2016/17; the estimates for 2017/18 onwards are based on HFS data (see main text). The HII series incorporates a top-income adjustment for years 2001/02 and thereafter based on the quantile method employing a 97% threshold and 0.5% quantile band width (see main text)

has applied its top-income adjustment, the HII series is above the HBAI series in all but two years. Again, this is what one would expect. If you make a more substantial correction for survey top-income under-coverage, your estimate of inequality increases relative to the status quo estimate, as BHJW also showed.

## 5 Discussion and conclusions

Although top-income adjustments to survey data drawing on income tax administrative data are currently in vogue among researchers, few know that the UK statistical agencies producing official income distribution statistics have been making such adjustments since 1992. The DWP was the pioneer; more recently, the ONS has extensively road-tested and now introduced its own top-income adjustment. It is natural to ask what the factors driving these initiatives were and whether other countries might follow the UK.

The *HBAI* data series was first published in 1988 (Department of Health and Social Security 1988b) and represented a major change in UK official statistics on low-income prevalence. The earlier official series, *Low Income Families*, first published in 1974, provided estimates of numbers of individuals and families with incomes below thresholds defined in terms of fractions of social assistance benefit rates.[10] In the *HBAI*, the thresholds are fractions of 'average' income (currently headline poverty statistics are based on a 60% of median income cut-off).[11]

The switch to *HBAI* followed the recommendations of a methodological review by an inter-departmental working group with national statistical office representation (DSS 1988a). Relatively soon after the *HBAI*'s introduction, there was a major stocktaking review by another inter-departmental working group, also involving external experts such as Tony Atkinson. This reported in November 1991: see DSS (1991), a 50-page report. The working group recommended the introduction of an SPI-adjustment though, interestingly the 2½ page discussion is under the heading of 'Treatment of Outliers' rather than referring to the dual concerns cited subsequently (see the Introduction). The recommendation was contingent: 'provided that a methodology can be established which commands the confidence of both DSS and external analysts as constituting an improvement on present practice' (DSS 1991: 12). Clearly, the conditions were met, and the rest is history as the saying goes, with some small modifications introduced subsequently (see BHJW 2018*a*).[12]

In sum, the conditions favouring the introduction of the SPI-adjustment were strong contemporary interest in the nature and quality of income distribution statistics,[13] combined with the willingness and ability of the department responsible to devote resources to substantial reviews and, also, to draw on advice from other relevant government departments and external analysts. Another factor that eased the introduction of the SPI-adjustment was that it was an incremental change and hence easier to implement than if it had been proposed as part of the initial introduction of the *HBAI* approach. Also, the adjustment was relatively simple and hence transparent and easy to explain.

A similar set of factors appears to have favoured the recent introduction of the ONS top-income adjustment. There remains substantial contemporary interest in income distribution statistics with a growing focus on inequality and those at the top of the distribution, accompanied by a more general interest in measuring 'well-being'. More specifically, the ONS was undertaking a broad-based review of household financial statistics; and the top-income adjustment work fell naturally within its scope. Resources were available to undertake extensive methodological work, as the ONS bulletins cited earlier demonstrate. Opportunities were taken to consult across government departments: the ONS top-income adjustment working group included a representative from the DWP's HBAI team. The group also consulted external analysts such as the author and drew heavily on academic research that provided not only arguments for modifications of existing arrangements but also demonstrations that they were feasible using existing UK data. The prior existence of the DWP's SPI-adjustment also helped. The ONS approach was arguably incremental in nature and, importantly, the change also represented a move towards greater consistency in approach across the two UK agencies that provide official income distribution statistics.

[10] The final edition in the series is Department of Health and Social Security (1988c). The DHSS was later split into the Departments of Health and of Social Security. Subsequently the DSS became the DWP.

[11] Johnson and Webb (1989) and Nolan (1989) review the changes from *LIF* to *HBAI*.

[12] Unfortunately, the full details of circumstances behind the DWP's introduction of the SPI-adjustment are lost in the mists of time: the relevant documentation are unavailable. However, I wish to salute the expert knowledge and wisdom of the then *HBAI* team leader, Gordon Harris. I suspect that the SPI-adjustment would not exist without his foresight and initiative.

[13] Remember too that the 1980s were when income inequality and poverty rates increased very substantially in the UK.

The importance of resources for methodological work on official statistics is illustrated by the fact that the DWP has not advertised plans to introduce a more extensive top-income adjustment, one more like BHJW's SPI2-adjustment or the ONS adjustment. I understand that the FRS and HBAI teams are smaller now than in the past. Perhaps more importantly, the DWP's priorities lie elsewhere at the time of writing. As a result of the Covid-19 pandemic, the Department is focusing on delivering support to a substantially larger caseload (and reallocated staff to do so). How to leverage real time information provided by administrative data on cash benefits and employment earnings is a greater research priority than a top-income adjustment modification. Relatedly, the DWP's principal responsibility in official statistics terms is for poverty statistics (and the ONS for inequality statistics). The headline poverty line is 60% of median income but the median and hence poverty incidence are unaffected by the top-income adjustment.

Are there lessons for academic researchers from the UK experience with top-income adjustments? One observation is that the top-income adjustment methods implemented by the DWP and the ONS are not as sophisticated as the state-of-the-art approach represented by, for instance, that of Blanchet et al. (2019) – though there are also questions about the robustness of the latter. (See, e.g., Flachaire et al. (2021) who raise questions specifically about the choice of top-income threshold differentiating the problematic group with high incomes from the rest of the population.) All three approaches involve choices about top-income thresholds, top-income group bandwidths, and attempt to adjust for top-income-related unit non-response and item non-response (under-reporting), but the DWP and ONS implementations are more ad hoc, and tailored to the specific applications to hand.[14] To an academic economist, these features might be seen as undesirable and, to be sure, the professional incentives are to introduce methods that are technically sophisticated and generally applicable (these are features that the leading journals value).

However, the same features are strengths in the context of official statistics and need to be considered alongside issues of methodological validity. Relative simplicity and transparency in statistical indicators and their derivation are important; so too is the ability to produce statistics to a regular frequency and in timely fashion – not as a one-off exercise as in much academic work.

In sum, in official statistical monitoring, the choice of a methodological approach requires trading off of multiple objectives in a way that academic economists are not used to. However, the positive lesson for academic economists is that if they are prepared to recognise this and are willing to engage closely with the relevant statistical agencies, they may help facilitate positive changes in official statistical practice. Tony Atkinson is a leading exemplar (see Brandolini et al. 2017 for illustrations of his influence on national and international statistical agencies).

Will statistical agencies in other countries introduce top-income adjustments to their survey-based income distribution statistics? The UK experience suggests that several factors need to co-exist for this to occur, including: income inequality and related distributional issues being salient issues in public policy discourse; resources available for methodological work; access to suitable administrative data; and the ability to continue producing inequality

[14] Interestingly, Blanchet et al.'s (2019, Figure 8) estimates for the UK bear close similarities to those shown in Figure 3 (the ONS estimates). That is, the Blanchet et al. 'replacing' approach (akin to the ONS quantile approach) yields Gini coefficient estimates that are only slightly smaller than their own method (a sophisticated version of the ONS reweighting approach). However, their application to Brazil shows much larger differences in estimates from the replacing method and their method, which underlies the country-specific nature of issues such as top-income under-coverage and how to address it.

estimates using standard definitions (such as the Canberra Group ones) and indices that are based on all incomes (notably the Gini coefficient).

In the USA, Bureau of Economic Analysis (BEA) research has explored top-income adjustments to the data from the Current Population Survey's Annual Social and Economic Supplement using income tax data from the Internal Revenue Service (Fixler et al. 2019). However, the ultimate goal of the project is to derive income distribution measures consistent with those in the national accounts (the BEA's responsibility). In the USA, the Census Bureau has charge of official income distribution statistics. Changes to these are possible, as illustrated by the introduction of the Supplemental Poverty Measure, which followed substantial background research (Citro and Michael 1985) and Census Bureau methodological work. However, as for the DWP in the UK, the Census Bureau's primary focus is on poverty statistics with inequality statistics a by-product. So, changes to US official poverty statistics are more likely than methodological innovations such as top-income adjustments that affect inequality statistics specifically.

The European Union illustrates constraints of a different kind. All member states contribute income distribution statistics and other social indicators to the pan-EU Statistics on Income and Living Conditions, coordinated and published by Eurostat. The deliverables, mandatory for all member states, are the result of a cross-national process of consultation and negotiation, the Open Method of Coordination (OMC). Although member states have some discretion about the instruments they use to collect and produce the mandated social indicators (e.g., household surveys or administrative registers), the likelihood of introducing a top-income adjustment by survey countries is small because the OMC procedure requires consensus.

By this point, some readers may be asking whether a reliance on household surveys coupled with a top-income adjustments is the right way to go in future – why not use administrative record (register) data more extensively to derive official income distribution statistics? If tax data are of good quality relative to household survey data, why use not them more extensively, i.e., not only for top incomes?

Relatively few countries can rely completely on administrative data – the leading examples are the Nordic countries, with a long history of using registers and, relatedly, societies that are comfortable with having unique personal identifiers and using them widely, including for cross-register linkages. Other countries have registers but greater concerns about privacy and national identity numbers mean that linked registers are not widely used (nor are the data available to non-governmental researchers). Moreover, statistical agencies are interested in the bottom half of the income distribution, not only the top, and income tax data – the data used for top-income adjustments – do not cover the bottom half well by comparison with household surveys. One needs to link multiple registers to get full distributional coverage. Regardless, many countries, especially poorer ones, do not have suitable registers and the use of household surveys as the foundation for their income distribution statistics is inevitable.

Another strategy is to retain the household survey but to link administrative records to survey respondents and replace survey responses with the administrative ones, as done by several countries participating in EU-SILC and for a range of income sources depending on the country (Carranza et al. 2021: 8–10).[15] Data substitution for cash transfers is less relevant if your concern is better accounting for top incomes when measuring inequality. Substitution of responses for other sources such as labour and capital incomes is more relevant, but relies on

[15] The *HBAI* introduced more extensive data substitution strategies for cash transfers in its latest edition. See DWP (2021*b*).

having suitable administrative data available. Nonetheless, this strategy assumes that the linked data are of better quality than the survey responses. This supposition is not necessarily correct. Incorrect linkages between survey and administrative records (matching the wrong people) can reduce data quality significantly. And not only survey responses, but also administrative record data, may contain measurement error depending on how the data are compiled, and this reduces the reliability of the linked administrative data as well.[16]

The overall conclusion I draw is that there are no general conclusions. The likelihood that a country will introduce a top-income adjustment into its survey-based income distribution statistical monitoring system is strongly contingent on country-specific circumstances. The UK's experience that I have described, pioneering as it is, may be unique.

**Acknowledgements** This paper was prepared for a special issue of the Journal of Economic Inequality on 'Finding the upper tail'. Many thanks to Richard Burkhauser, Nicholas Hérault, and Roger Wilkins. I have drawn heavily on our joint work (especially Burkhauser et al. 2018a, 2018b) in Sections 2 and 3 but they are not responsible for how I have done so. Richard Burkhauser and Nora Lustig provided helpful comments on earlier drafts.

## Declarations

**Competing Interests** The author has no competing interests to declare that are relevant to the content of this article.

**Open Access** This article is licensed under a Creative Commons Attribution 4.0 International License, which permits use, sharing, adaptation, distribution and reproduction in any medium or format, as long as you give appropriate credit to the original author(s) and the source, provide a link to the Creative Commons licence, and indicate if changes were made. The images or other third party material in this article are included in the article's Creative Commons licence, unless indicated otherwise in a credit line to the material. If material is not included in the article's Creative Commons licence and your intended use is not permitted by statutory regulation or exceeds the permitted use, you will need to obtain permission directly from the copyright holder. To view a copy of this licence, visit http://creativecommons.org/licenses/by/4.0/.

## Data Availability

The data used in Table 1 and Figs. 1 and 2 and how to access them are described in the replication materials for Burkhauser et al. (2018b). The sources of the data used in Figs. 3 and 4 are the publicly available spreadsheets cited in the notes to the figures.
